# Identification of a novel cholesterol-lowering dipeptide, phenylalanine-proline (FP), and its down-regulation of intestinal ABCA1 in hypercholesterolemic rats and Caco-2 cells

**DOI:** 10.1038/s41598-019-56031-8

**Published:** 2019-12-19

**Authors:** Arata Banno, Jilite Wang, Kenji Okada, Ryosuke Mori, Maihemuti Mijiti, Satoshi Nagaoka

**Affiliations:** 1 Department of Applied Life Science, Faculty of Applied Biological Sciences, Gifu University, 1-1 Yanagido, Gifu, 501-1193 Japan; 2 Department of Agriculture, College of Hetao, Bayannur, 015000 China

**Keywords:** Peptides, Peptides, Dyslipidaemias, Dyslipidaemias

## Abstract

There has been no report about *in vivo* active cholesterol-lowering dipeptide in any protein origin, despite their potential health benefits. Cattle heart protein hydrolysate ultra-filtrate (HPHU, molecular weight < ca. 1,000 Da peptide mixture) exhibits cholesterol-lowering activity in hypercholesterolemic rats, but the active peptide in HPHU that lowers serum cholesterol levels and its molecular mechanism are unknown. In this study, we separated and purified HPHU to identify a novel cholesterol-lowering dipeptide (phenylalanine-proline, FP) and characterized the mechanism underlying its effects *in vivo* and *in vitro*. We identified FP as an active peptide from HPHU by MALDI-TOF mass spectrometry. FP significantly decreased serum total and non-HDL cholesterol and hepatic cholesterol levels in rats. FP significantly increased serum HDL cholesterol, accompanied by a significant decrease in the atherogenic index. FP also significantly increased fecal cholesterol and acidic steroid excretion. Moreover, FP significantly decreased ATP-binding cassette transporter A1 (ABCA1) expression in the rat jejunum and reduced cholesterol absorption in Caco-2 cells. We found a novel cholesterol-lowering dipeptide FP that could improve cholesterol metabolism via the down-regulation of intestinal ABCA1. The cholesterol-lowering action induced by FP was disappeared in *PepT1*KO mice. FP-induced cholesterol-lowering action is mediated via PepT1 in mice.

## Introduction

Increased serum total cholesterol and LDL cholesterol and decreased HDL cholesterol induces hypercholesterolemia, which is a critical risk factor for atherosclerosis and ischemic heart disease^1,2^. Therefore, the maintenance of proper cholesterol levels in the serum is essential for good health. The primary strategy for the prevention of disorders associated with hypercholesterolemia is dietary modification, including foods or dietary components with functional properties. Many researches using experimental animals and humans have suggested that vegetable proteins, such as soybean protein, can significantly decrease the risk of atherosclerosis by reducing plasma lipids^3–5^. Furthermore, recent studies have confirmed that various animal proteins, such as milk whey^6,7^ and egg white protein^8,9^, have hypocholesterolemic effects. It has been reported that peptides or protein hydrolysates show greater bioactivity than that of intact proteins. Bioactive peptides have been produced *in vitro* by the chemical or enzymatic hydrolysis of several dietary proteins to modify and improve the physiological functions^7^. However, few studies have examined the effects of animal proteins and animal protein hydrolysates on cholesterol metabolism.

Cholesterol is a water-insoluble molecule; its intestinal absorption is complex, similar to other lipids, including a micellar solubilization step^10^. The modulation of intestinal cholesterol absorption by dietary protein and other food constituents may explain the cholesterol-lowering effects of foods. Some studies have suggested that dietary proteins, such as soybean protein^11,12^ and sunflower protein hydrolysates^13^ decrease the micellar solubility of cholesterol *in vitro* and have hypocholesterolemic actions in animals. Very little is known about specific food-derived peptides that reduce serum cholesterol levels *in vivo* and hence more researches are required in this field.

We previously reported that cattle heart protein hydrolysate (HPH) and cattle heart protein hydrolysate ultra-filtrate (HPHU, MW < ca. 1,000 Da peptide fraction) exert strong hypocholesterolemic activity in rats^14^. HPH significantly reduces the serum total cholesterol and atherogenic index. HPH also significantly increases the fecal output of total steroids and cholesterol. The reduction of cholesterol uptake into Caco-2 cells was significantly greater in cholesterol micelles containing HPH than in the cholesterol micelles containing casein. The micellar solubility of cholesterol was significantly lower in the presence of HPH than in the presence of casein. Moreover, HPHU derived from HPH had a higher ability to reduce both serum and liver cholesterol concentrations than HPH in rats. We speculate that HPHU contains an active cholesterol-lowering peptide and a specific active peptide must play a pivotal role in the cholesterol-lowering actions of HPHU. However, the active peptide in HPHU and the mechanism underlying its cholesterol-lowering effect is unknown.

As about 80% of digested proteins are absorbed in the form of dipeptides or tripeptides, Proton-coupled oligopeptide transporter 1 (PepT1, Slc15A1) has an essential function in protein assimilation^15^. PepT1 is a highly conserved transporter that is existed in various mammalian species. PepT1mediates the uptake of di- and tripeptides in the intestine and kidney. *PepT1* KO mice had generated to further clarify the physiological significance of existence of specific peptide transporters in the intestine^15^. *PepT1* deletion drastically decreased the intestinal uptake of dipeptide GlySar, and its oral absorption by gastric intubation. There has been no report about the relationship between PepT1 and cholesterol-lowering action induced by dietary peptides and proteins. With the use of wildtype (WT) and *PepT1* knockout (KO) mice, this study provided definitive evidence for the FP induced cholesterol-lowering action.

In this study, we separated and purified HPHU by size exclusion chromatography (SEC) and reversed-phase chromatography (RPC) to identify a novel cholesterol-lowering peptide. After the identification of an active peptide, we evaluated the mechanism underlying its cholesterol-lowering action *in vivo* including *PepT1*KO mice and *in vitro*.

## Results

### Purification of the inhibitory peptide for cholesterol micellar solubility *in vitro*

**Figure 1:**
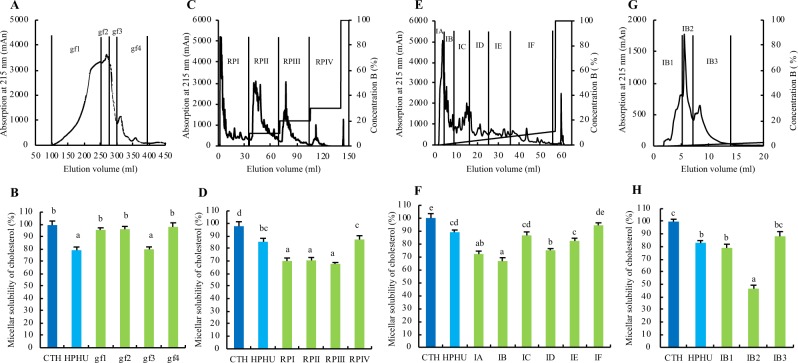
Peptide purification and the effects of HPHU fractions, HPHU or CTH on the micellar solubility of cholesterol *in vitro*. (**A**) Typical elution profiles of HPHU by HPLC. Elution profile of HPHU by SEC. HPHU was dissolved in 10 mL of Milli Q water and applied to the column. The fractions gf1–gf4 were collected and freeze-dried. (**B**) The effects of gf1–gf4 fractions on the micellar solubility of cholesterol *in vitro*. (**C**) Typical elution profiles of gf3 by HPLC. Elution profile of gf3 by RPC using 1 mL of gf3 solution (10 mg of protein) in 2% acetonitrile including 0.065% trifluoroacetic acid (TFA). (**D**) Effect of the gf3 fraction on the micellar solubility of cholesterol *in vitro*. (**E**) Typical elution profiles of RPI by HPLC. Elution profile of RPI by RPC using 1 mL of RPI solution (10 mg protein) in 2% acetonitrile including 0.065% TFA. (**F**) Effect of RPI fractions on the micellar solubility of cholesterol *in vitro*. (**G**) Typical elution profiles of IB by HPLC. Elution profile of IB by RPC using 1 mL of IB solution (10 mg protein) in 2% acetonitrile including 0.065% TFA. (**H**) Effect of IB fractions on the micellar solubility of cholesterol *in vitro*. Values are means with standard errors represented by vertical bars (n = 4 per group). Different superscripts indicate significantly differences determined by Tukey’s test (P < 0.05).

The typical elution profile of HPHU by a HiLoad 26/60 Superdex 30 pg column is shown in Fig. 1A. Peaks (1−4) were consistently detected in separate experiments. The fractions gf1–gf4 were collected and the effect of these fractions on cholesterol micellar solubility *in vitro* were evaluated as shown in Fig. 1B. The fractions gf1, gf2, gf3, and gf4 accounted for approximately 23.6%, 17.4%, 14.8%, and 1.4% of HPHU, respectively. The cholesterol micellar solubility of HPHU (79.1%) or gf3 (79.9%) was significantly lower than those of CTH (control) or other fractions, like gf1 (95.6%), gf2 (96.2%), or gf4 (98.0%), as shown in Fig. 1B. These results suggested that active peptides of HPHU related to the inhibitory effect on cholesterol micellar solubility are concentrated in the gf3 fraction.

The typical RPC elution profile of gf3 is shown in Fig. 1C. The gf3 fraction was separated into RPI (0% B), RPII (0–10% B), RPIII (10–20% B), and RPIV (20–30% B) and the effect of these fractions on the micellar solubility of cholesterol was evaluated *in vitro*. The fractions RPI, RPII, RPIII, and RPIV accounted for approximately 65.0%, 14.5%, 3.0%, or 0.75% of gf3, respectively. RPI (70.1%), RPII (70.6%), and RPIII (67.8%) showed lower cholesterol micellar solubilities than RPIV (87.2%), as shown in Fig. 1D.

The typical RPC elution profile of RPI is shown in Fig. 1E. RPI was separated into six fractions, IA–IF, and the effects of the fractions on the micellar solubility of cholesterol were evaluated *in vitro*. The fractions IA, IB, IC, ID, IE, or IF accounted for approximately 39.0%, 12.7%, 4.0%, 6.7%, 4.1%, and 2.4% of RPI, respectively. IA (72.3%) and IB (66.8%) showed lower cholesterol micellar solubilities than IC (86.7%), ID (75.2%), IE (82.4%), and IF (94.5%), as shown in Fig. 1F.

The typical RPC elution profile of IB is shown in Fig. 1G. IB was separated into four fractions, IB1–IB3, and the effects of the fractions on the micellar solubility of cholesterol were evaluated *in vitro*. The fractions IB1, IB2, and IB3 accounted for approximately 12.8%, 13.4%, or 10.5% of the IB fraction, respectively. IB2 (46.6%) showed lower cholesterol micellar solubility than IB1 (78.9%) or IB3 (88.1%), as shown in Fig. 1H.

### Molecular weight distribution of IB2 containing peptides estimated by gel filtration

**Figure 2:**
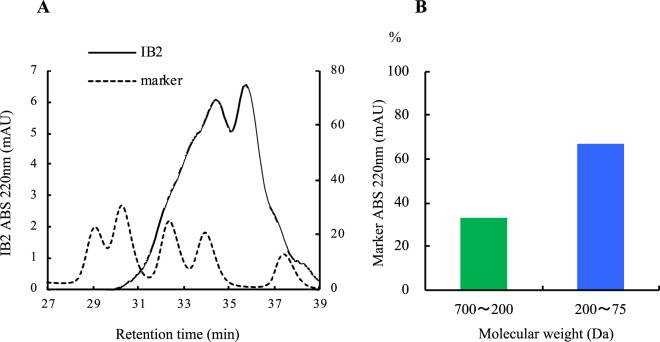
Molecular weight (M.W.) distribution of IB2 containing peptides estimated by gel filtration. (**A**) Typical elution profiles of IB with molecular weight standards by HPLC. (**B**) Molecular weight (M.W.) distribution (%) of IB2 containing peptides estimated by gel filtration.

IB2 contained the peptides having a molecular weight < 700 was estimated by gel filtration using Superdex Peptide 10/300 GL (Fig. 2A). Peptide molecular weight 700~200 or 200~75 was contained in IB2 fraction 33% or 67% respectively by calculating the area of elution profile as shown in Fig. 2B.

### Identification of an active peptide (FP) in IB2

**Figure 3:**
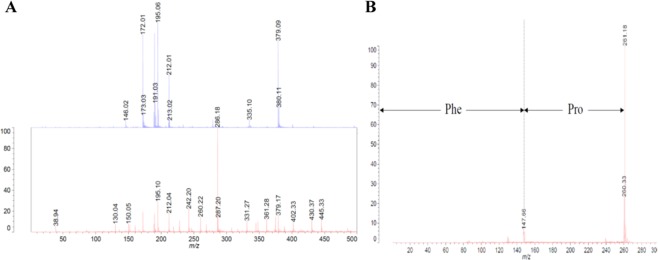
Identification of an active peptide (FP) in IB2 by MALDI-TOF/MS analysis. (**A**) The peptide in the fraction IB2 was analyzed by MALDI-TOF mass spectrometry. Upper: matrix blank, Lower: IB2 (**B**) MS/MS analysis of 261.18 m/z.

The MALDI-TOF/MS spectrum revealed that IB2 contained some peptides, including FP (261.18 m/z) as shown in Fig. 3A. FP was identified by MS/MS analysis (Fig. 3B). FP can be released from Myosin-2 or Actin (alpha-cardiac muscle), the major cattle heart protein, and their position within Myosin-2 (Q9BE41) from N to C terminal: 78–79, 543–544, 714–715 or Actin (Q3ZC07) from 43–44 in data bases.

### Effect of CTH, HPHU or FP on cholesterol micellar solubility *in vitro* and cholesterol absorption in Caco-2 cells

**Figure 4:**
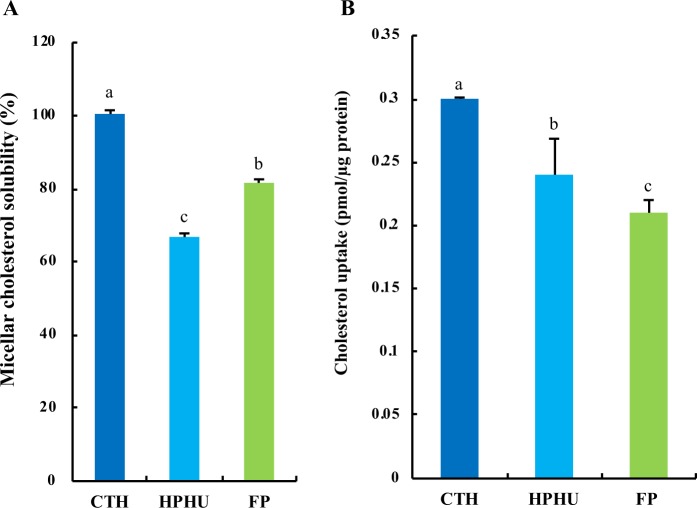
Effects of HPHU, CTH or FP on cholesterol micellar solubility *in vitro* and cholesterol absorption in Caco-2 cells. (**A**) Effects of CTH, HPHU or FP on micellar solubility of cholesterol *in vitro*. Values are means with standard errors represented by vertical bars (n = 4 per group). Different superscripts indicate significantly differences determined by by Tukey’s test (P < 0.05). (**B**) After Caco-2 cells were treated with [^14^C]-labeled micellar solutions (0.5 mL) containing CTH (5 g/L), HPHU (5 g/L) or FP (5 g/L) respectively, and the cell lysates were collected. The amount of cholesterol absorbed by cells is expressed as p mol/mg protein. Values are means, with standard errors represented by vertical bars (n = 6 per group). Different superscripts indicate significantly differences determined by Tukey’s test (P < 0.05).

Micellar solubility of cholesterol *in vitro* was significantly decreased by HPHU or FP compared with CTH (Fig. 4A) To address whether HPHU or FP can inhibit cholesterol absorption, Caco-2 cells were treated with micelle containing FP, HPHU or CTH. Cholesterol uptake from the micelle with FP or HPHU (5 g/L) was significantly lower than that from the micelle containing CTH (Fig. 4B).

### Effect of FP on the mRNA levels of cholesterol metabolism-related genes in Caco-2 cells

**Figure 5:**
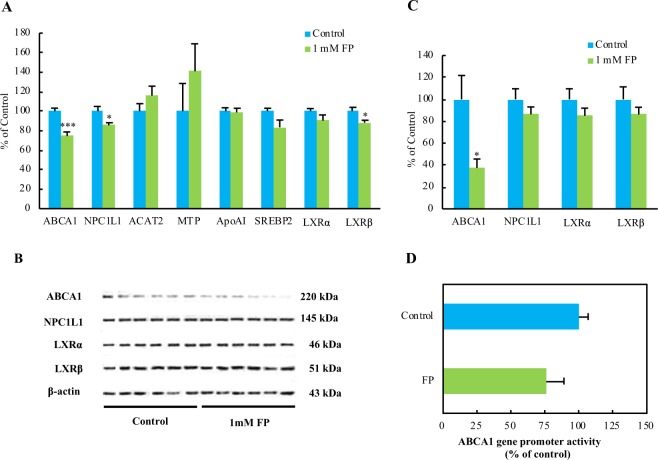
Effects of FP on the expression of cholesterol metabolism-related genes and ABCA1 promoter activity in Caco-2 cells. After Caco-2 cells were treated with vehicle control or 1 mM FP for 24 h, total RNA and cell lysates were collected. (**A**) ABCA1, NPC1L1, ACAT2, MTP, ApoAI, SREBP2, LXRα, and LXRβ mRNA levels were measured by real-time PCR and normalized to the mRNA expression level of 18 s ribosomal RNA. (**B**) ABCA1, NPC1L1, LXRα, and LXRβ protein levels were analyzed by western blotting. (**C**) Summary of ABCA1, NPC1L1, LXRα, and LXRβ protein levels normalized to the level of β-actin. (**D**) Caco-2 cells were transfected with the human pGK3-ABCA1-Luc plasmid, using the pGK β-galactosidase plasmid as an internal control. After transfection, the cells were treated with 1 mM FP for 12 h. Data are presented as luciferase activity normalized to β-galactosidase activity. Values are means, with standard errors represented by vertical bars (n = 6 per group). Asterisks indicate differences from the control (*P < 0.05, ***P < 0.001) determined by Student’s *t*-tests.

To investigate the effect of FP on the mRNA levels of cholesterol metabolism-related genes in Caco-2 cells, we performed real-time PCR using Caco-2 cells treated with or without 1 mM FP for 24 h. We investigated whether FP affected the mRNA levels of ABCA1, Niemann-pick C1-like 1 L1 (NPC1L1), Acyl-coenzyme A: cholesterol acyltransferase 2 (ACAT2), Microsomal triglyceride transfer protein (MTP), Apolipoprotein AI (ApoAI), Sterol regulatory element binding protein 2 (SREBP2), Liver X receptor α (LXRα), and Liver X receptor β (LXRβ). The addition of FP significantly decreased the mRNA levels of ABCA1 and NPC1L1 (Fig. 5A). Recent studies have shown that SREBP and LXR are important transcription factors that regulate ABCA1 and NPC1L1 gene transcription. FP significantly decreased the LXRβ mRNA level (Fig. 5A). In addition, SREBP2 and LXRα mRNA levels tended to be lower in FP-treated group than in the control group (Fig. 5A).

### Effect of FP on ABCA1, NPC1L1, and LXR protein levels in Caco-2 cells

To evaluate the relationships between mRNA and protein levels, we further evaluated the protein levels of ABCA1, NPC1L1, LXRα, and LXRβ in Caco-2 cells. FP significantly decreased the ABCA1 protein level (Fig. 5B,C). However, FP did not affect NPC1L1, LXRα, and LXRβ levels (Fig. 5B,C).

### Effect of FP on *ABCA1* promoter activity in Caco-2 cells

We investigated the effect of FP on ABCA1 gene promoter activity. Caco-2 cells were treated with or without 1 mM FP for 12 h, and the cell lysates were subjected to a luciferase assay. FP did not significantly affect ABCA1 promoter activity (Fig. 5D).

### Effect of FP on metabolic parameters in rats fed a high-fat high-cholesterol diet

**Figure 6:**
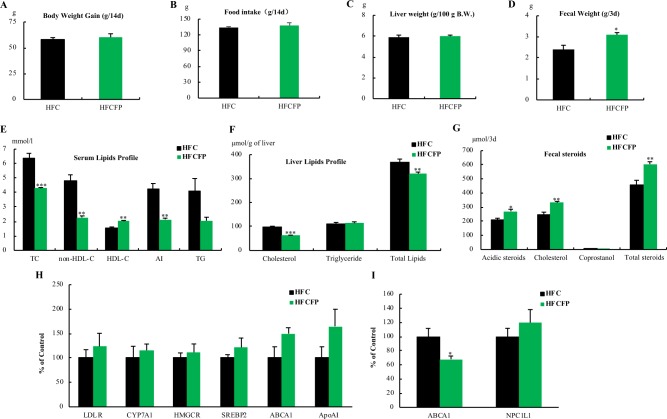
Effects of dietary FP on metabolic parameters and the expression of cholesterol metabolism-related genes in rats fed a high-fat high-cholesterol diet. (**A**) Body weight gain, (**B**) Food Intake, (**C**) Liver weight, (**D**) Fecal weight, (**E**) Serum lipids profile, non-HDL-cholesterol = Total cholesterol-HDL cholesterol, Atherogenic index (AI) = Total cholesterol/HDL cholesterol, (**F**) Liver lipids profile, (**G**) Fecal steroids, Total steroids = acidic steroids + cholesterol + coprostanol. (**H**) Effects of the oral administration of FP on hepatic mRNA levels of genes related to cholesterol metabolism in rats. (**I**) Effects of the oral administration of FP on jejunal mRNA levels of genes related to cholesterol metabolism in rats. Values are means, with standard errors represented by vertical bars (n = 5 per group). Asterisks indicate differences from the Control group (*P < 0.05, **P < 0.01, ***P < 0.001) determined by Student’s *t*-tests.

The body weight gain, total food intake, and liver weight were unaffected by dietary treatment over 14 days (Fig. 6A–G). The serum total and non-HDL-cholesterol concentrations in the HFCFP (High Fat and High Cholesterol diet fed FP: FP group) group were significantly lower than those in the HFC (High Fat and High Cholesterol diet without FP: Control group) group. The serum HDL cholesterol level was significantly higher in the HFCFP group than in the HFC group, resulting in a significant decrease in the atherosclerosis index. The serum triglyceride level tended to be lower in the HFCFP group than in the HFC group. Liver cholesterol and liver total lipids in the HFCFP group were significantly lower than those in the HFC group. The levels of fecal steroid excretion of acidic steroids and cholesterol in the HFCFP group were significantly higher than those in the HFC group.

### Effect of FP on the mRNA levels of cholesterol metabolism-related genes in rats

We evaluated the effect of FP on the mRNA levels of cholesterol metabolism-related genes in rats. FP did not significantly affect the hepatic mRNA expression of genes related to cholesterol metabolism such as low density lipoprotein receptor (LDLR), cholesterol 7α-hydroxylase (CYP7A1), 3-hydroxy-3-methylglutaryl coenzyme A reductase (HMGCR), SREBP2 or ApoAI, but tended to increase ABCA1 mRNA expression related to HDL formation (P = 0.088, 150%) (Fig. 6H). In contrast, FP induced a decrease in the ABCA1 mRNA level in the jejunum related to the inhibitory action of intestinal cholesterol absorption (Fig. 6I).

### Effect of FP on metabolic parameters and cholesterol absorption in *PepT1* knockout (KO) and wildtype (WT) mice fed a high-fat high-cholesterol diet

**Figure 7:**
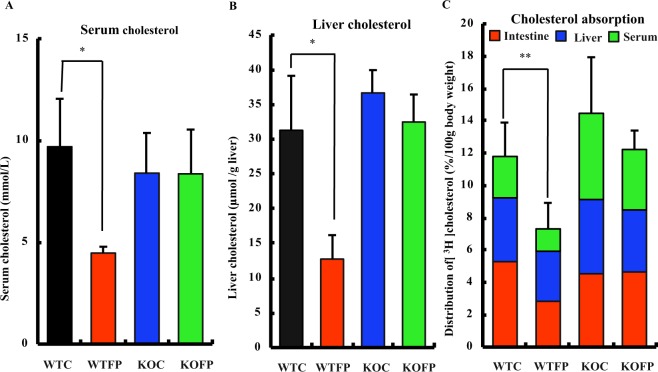
Effects of FP on serum and liver cholesterol, cholesterol absorption in wildtype (WT) and *PepT1* knockout (KO) mice fed a high-fat high-cholesterol diet. (**A**) Serum cholesterol, (**B**) Liver cholesterol, (**C**) Cholesterol absorption. Values are means, with standard errors represented by vertical bars (n = 8 or 9 per group). Statistical significance compared with the control group (WTC vs WTFP) by Student’s t-test (*P < 0.05, **P < 0.01).

The body weight gain was significantly increased in WTFP group compared to WTC group, and KOFP group compared to KOC group. Food intake was significantly increased in KOFP group compared to KOC group. Liver weight was significantly decreased in WTFP compered to WTC group (Supplementary Table 1). Serum cholesterol in WTFP group was significantly lower than that in WTC group (Fig. 7A). Liver cholesterol (Fig. 7B) and liver total lipids (Supplementary Table 1) in WTFP group were significantly lower than those in the WTC group and were not significantly changed among KO groups. Cholesterol absorption was significantly decreased in WTFP compared to WTC and was not significantly changed among KO groups (Fig. 7C).

## Discussion

FP is the world’s first cholesterol-lowering dipeptide. This is the first study to identify FP as a novel cholesterol-lowering dipeptide. We previously reported that HPHU exhibits a cholesterol-lowering effect *in vivo* and *in vitro*^14^. However, the active peptide of HPHU and the mechanisms by which the active peptide induces a hypocholesterolemic effect were unknown. In the present study, in addition to identifying FP, we evaluated its cholesterol-lowering action *in vivo* and *in vitro*.

In particular, we evaluated the inhibitory effect of HPHU fractions on cholesterol micellar solubility and identified the peptide sequences. Several studies have suggested that suppressed cholesterol micellar solubility is related to the inhibitory action of cholesterol absorption in Caco-2 cells and *in vivo*. It is well known that the human epithelial cell line Caco-2 has been widely used as a model of the intestinal epithelial cells^16^. For example, HPHU, soy protein peptic hydrolysate (SPH), and β-lactoglobulin tryptic hydrolysate (LTH) have inhibitory effects on both cholesterol micellar solubility and cholesterol absorption in Caco-2 cells and are associated with a negative correlation between serum total cholesterol and fecal total steroid excretion *in vivo*^7,11,14,17,18^. Therefore, we speculated that the active peptides in HPHU had inhibitory effects on cholesterol micellar solubility and cholesterol absorption in Caco-2 cells, accompanied by a negative correlation between serum total cholesterol and fecal total steroid excretion. Thus, we identified an active dipeptide, FP by evaluations of cholesterol micellar solubility and cholesterol absorption in Caco-2 cells *in vitro*. However, FP-containing fraction is not the only one carrying cholesterol-lowering properties (Fig. 1). Moreover, FP peptide may not be the only peptide in the fraction that has hypocholesterolemic properties (Fig. 3). We will be going to clarify the active peptide other than FP to evaluate whether the cholesterol-lowering action of HPHU is due to FP peptide or other peptides. Also, we need to clarify the contribution of FP on the cholesterol-lowering action of HPHU by preparing FP-free HPHU in future study.

Moreover, we demonstrated that orally administrated FP results in significant reductions in serum total and non-HDL-cholesterol concentrations in hypercholesterolemic rats fed a high-fat high-cholesterol diet (Fig. 7E). This result suggests that FP, a cholesterol-lowering peptide, is directly responsible for the hypocholesterolemic effects of HPHU. In the present study, the high level of serum HDL cholesterol in response to FP treatment indicate that FP results in a significant decrease in the atherogenic index (total cholesterol/HDL-cholesterol), which may result in the transfer of peripheral free cholesterol to the liver by a mechanism known as “reverse cholesterol transfer,” promoted by HDL cholesterol.

The blood cholesterol level is determined by cholesterol absorption, synthesis, storage, degradation, and excretion^19^. In our previous study, we found a significant correlation between fecal total steroids excretion and serum total cholesterol in rats^11^. Thus, our data suggested that the fecal excretion of bile acids and cholesterol could be affected by FP feeding. FP induced a significant increase in the fecal excretion of bile acids and cholesterol compared to levels in the control. Thus, the cholesterol-lowering action of FP inhibited intestinal cholesterol absorption and stimulated fecal steroid excretion.

Furthermore, to investigate the mechanism by which FP treatment decreased serum total and non-HDL-cholesterol concentrations in rats, we evaluated the effects of FP on cholesterol metabolism-related genes in rats and Caco-2 cells as a model of the small intestine. FP did not significantly affect hepatic mRNA expression of genes related to cholesterol metabolism, but tended to increase hepatic ABCA1 mRNA expression in rats (Fig. 6H). FP resulted in a significant decrease in ABCA1 mRNA levels in the jejunum (Fig. 6I). Furthermore, we clarified that FP induced a significant decrease in the ABCA1 mRNA level accompanied by a down-regulation of ABCA1 protein levels in Caco-2 cells (Fig. 5). These results related to Caco-2 cells are consistent with the results of *in vivo* rat experiments about jejunal ABCA1. FP did not affect cell growth measured by WST-1 method up to 1 mM in Caco-2 cells. We need to measure how much FP is detected in the stomach and gut content following HPHU supplementation in rats to optimize the effects of FP on cholesterol metabolism in Caco-2 cells in future study.

In addition, we found that cholesterol absorption decreased significantly in response to FP treatment in Caco-2 cells (Fig. 4B). NPC1L1 has a critical role in the intestinal absorption of dietary cholesterol, and the inhibition of NPC1L1 expression has a protective effect against hypercholesterolemia^20,21^. Our results showed that FP induced a significant decrease in NPC1L1 mRNA expression did not significantly affect NPC1L1 expression at the protein level (Fig. 5). Therefore, we speculate that the effect of FP on cholesterol absorption does not involve NPC1L1 expression.

ABCA1 mediates HDL cholesterol biogenesis by promoting the efflux of cholesterol and phospholipids to ApoAI^22^. ABCA1 is widely expressed throughout the body. However, studies have shown that hepatic and intestinal ABCA1 explain ~30% of plasma HDL cholesterol levels^22^. Recent studies have shown that cholesterol absorption in intestinal ABCA1 KO mice is reduced by ~28% and total plasma cholesterol is reduced by approximately 30%^23^. It is possible that the effect of FP on cholesterol absorption involves the down-regulation of ABCA1 expression. Though a truly useful intestinal ABCA1 specific inhibitor to decrease blood cholesterol has not yet to be discovered, peptides should be a key molecule to finding one.

The expression of ABCA1 is regulated by ABCA1 gene transcription and mRNA stability. ABCA1 gene transcription is mainly regulated by liver X receptors (LXRs). Excess cholesterol accumulation induces ABCA1 expression via LXRs^24,25^. We found that FP induces a significant decrease in the LXRβ mRNA level but did not affect LXRβ protein levels in Caco-2 cells (Fig. 5). Furthermore, we found that the down-regulation of ABCA1 mRNA was not caused by a decrease in ABCA1 transcriptional activity (Fig. 6). Thus, we speculate that the down-regulation of ABCA1 may be related to a decrease in mRNA stability by FP. We need to clarify the detailed mechanism about ABCA1 downregulation induced by FP in rats in future study. Recent studies have clarified that ABCA1 mRNA stability is regulated by micro-RNAs and RNA binding proteins^26–31^. In future studies, it is necessary to evaluate the effects of FP on miRNA and RNA binding protein profiles.

While, apoE mimetic peptides have increased potency to reduce plasma cholesterol through the changes of electrophoretic mobility of LDL particle in mice^32^. We tried to experimentally evaluate the effect of FP on the electrophoretic mobility of LDL particle *in vitro*. Our results suggested that FP did not affect the electrophoretic mobility of LDL particle *in vitro*. Thus, we suggest that the mechanism of cholesterol-lowering action of FP is different from apoE mimetic peptides.

**Figure 8:**
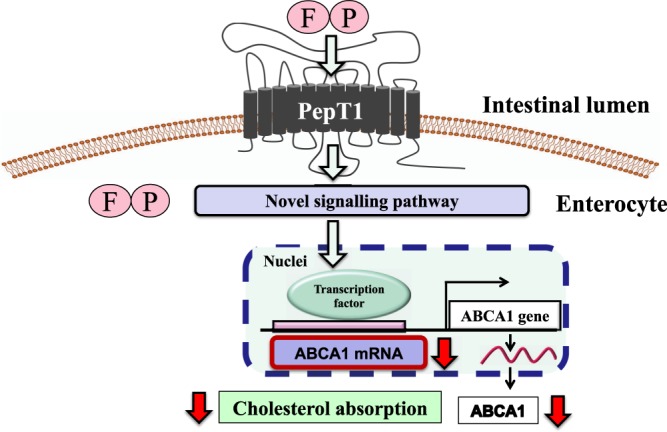
Mechanism of FP induced cholesterol-lowering action via a suppression of intestinal ABCA1 gene expression.

We had already discovered an *in vivo* active novel cholesterol-lowering pentapeptide, IIAEK (lactostatin) mediated the novel Ca-channel related Mitogen Activated Protein Kinase (MAPK) pathway for cholesterol degradation in human hepatocytes^33,34^. We speculate that lactostatin induce cholesterol degradation mediated a novel pathway via a hypothetical membrane lactostatin receptor (receptor hypothesis) and dipeptides, such as EK, mediate a novel pathway via a non-membrane receptor (the proton-coupled oligopeptide transporter 1 (PepT1) mediated pathway (non-receptor hypothesis)^34,35^. As FP can enter into Caco-2 cells through PepT1, we speculate that FP may mediate a novel pathway via a non-membrane receptor in the intestinal cell. Interestingly, FP-induced the cholesterol-lowering action was disappeared in *PepT1*KO mice. Thus, this shows that FP-induced cholesterol-lowering action is mediated via PepT1 in mice (Fig. 8) This clearly suggests that PepT1 is a crucial target for the improvement of cholesterol metabolism.

While, soybean glycinin derived three kinds of peptides (IAVPGEVA, IAVPTGVA, and LPYP) that are able to activate the low-density lipoprotein (LDL) receptor−SREBP2 pathway in HepG2 cells *in vitro*^36^. Thus, we need to clarify the molecular action of *in vivo* active cholesterol-lowering oligopeptides including IIAEK mediated via a membrane receptor or a non-membrane receptor in the future.

In summary, we identified the novel hypocholesterolemic dipeptide FP from HPHU by MALDI-TOF mass spectrometry as well as *in vitro* and *in vivo* assays related to cholesterol metabolism. Hypocholesterolemic dipeptides derived from proteins have not been characterized. FP significantly reduced serum total cholesterol, non-HDL-cholesterol, and hepatic cholesterol concentrations in hypercholesterolemic rats. FP significantly increased serum HDL cholesterol, resulting in a significant decrease in the atherogenic index. FP also significantly increased the fecal excretion of acidic steroids and cholesterol. Our results show that FP reduces intestinal cholesterol absorption via the down-regulation of ABCA1 expression. There is no scientific principle about the relationship between the structure (including amino acid sequence) and activity of cholesterol-lowering action. We need to clarify this unknown principle to find new peptides and promote the development of new functional foods and atherogenic drugs. Thus, these novel findings about FP play a crucial role in both the study of the effect of peptides on cholesterol metabolism and new functional foods and atherogenic drugs.

## Methods

### Preparation of the cattle heart protein hydrolysate ultra-filtrate (HPHU)

HPHU was provided by Itoham Foods Inc. (Ibaraki, Japan). Samples were prepared as described in our previous study^14^. HPHU consisted of 81.3% protein, 3.5% sugar, 0.4% lipids, 7.7% ash, and 7.1% moisture.

### Purification of the inhibitory peptide for cholesterol absorption or cholesterol micellar solubility *in vitro*

HPHU was separated according to the procedure described previously with some modifications. SEC was performed using a HiLoad 26/60 Superdex 30 pg Column (GE Healthcare) and the AKTA Avant 25 system (GE Healthcare). HPHU (0.57 g) was dissolved in 10 mL of deionized water and applied to the column. The flow rate was 2.0 mL/min. Online UV absorbance was monitored at 215 nm. The typical elution profile was shown in Fig. 1A. The eluted fractions (gf1~gf3) were collected and the effect on cholesterol micellar solubility was evaluated *in vitro*.

The fraction with the greatest inhibitory effect on cholesterol micellar solubility was collected (gf3), lyophilized, and further fractionated by RPC using a column (SOURCE 5RPC ST 4.6/150; GE Healthcare). The conditions for RPC were as follows. Solvent A was 0.065% trifluoroacetic acid (TFA) in 2% acetonitrile and solvent B was 0.05% TFA in 80% acetonitrile. The flow rate was 1 mL/min. The solvent gradient was initially 0% B for 35 min, then changed from 0% to 10% B for 35 min, from 10% to 20% B for 35 min, from 20% to 30% B for 35 min, and from 30% to 100% B for 47.5 min. Online UV absorbance was monitored at 215 nm. The typical elution profile was shown in Fig. 1C. The fractions (RPI~RPIV) were collected and the effect of these fractions on cholesterol micellar solubility was evaluated *in vitro*.

The fraction (RPI) with the greatest inhibitory effect on cholesterol micellar solubility was collected, lyophilized, and further fractionated by RPC using the same column described above. The conditions for RPC were as follows. Solvent A was 0.065% TFA in 2% acetonitrile and solvent B was 0.05% TFA in 80% acetonitrile. The solvent gradient was initially 0% B for 2.5 min, then changed from 0% to 11% B for 55 min and from 11% to 100% B over 10 min. Online UV absorbance was monitored at 215 nm. The typical elution profile was shown in Fig. 1E. The fractions (IA~IF) were collected and the effect of these fractions on cholesterol micellar solubility was evaluated *in vitro*.

The fraction (IB) with the greatest inhibitory effect on cholesterol micellar solubility was collected, lyophilized, and further fractionated by RPC using the same column described above. The conditions for RPC were as follows. Solvent A was 0.065% TFA in 2% acetonitrile and solvent B was 0.05% TFA in 80% acetonitrile. The solvent gradient was initially 0% B for 2.5 min, then changed from 0% to 4% B for 25 min and from 11% to 100% B over 12.5 min. Online UV absorbance was monitored at 215 nm. The typical elution profile was shown in Fig. 1G. The fractions (IB1~IB3) were collected and the effect of these fractions on cholesterol micellar solubility was evaluated *in vitro*.

### Effects of fractions derived from HPHU, CTH or FP on cholesterol micellar solubility *in vitro*

The effects of HPHU fractions, CTH or FP on cholesterol micellar solubility were evaluated *in vitro* using a previously described method^11,37^. The [4-^14^C]-labeled micellar solutions (1.0 mL) containing the following components were mixed by sonication (Ultrasonic Homogenizer, Model VP-5; Taitec, Tokyo, Japan): 0.74 kBq [4-^14^C]-cholesterol (2.1 Gbq/mmol; PerkinElmer Life Sciences, Yokohama, Japan), 0.1 mM cholesterol (Sigma Aldrich, St. Louis, MO, USA), 6.6 mM sodium taurocholate (Sigma Aldrich), 1 mM oleic acid (Sigma Aldrich), 0.6 mM phosphatidylcholine (Sigma Aldrich), 0.5 mM monoolein (Sigma Aldrich), 132 mM NaCl, and 15 mM sodium phosphate buffer (pH 7.4). After incubation at 37 °C for 24 h, the sample (5 g/L, respectively) was added to the micellar solution, solubilized by sonication, incubated at 37 °C for 1 h, and centrifuged at 100,000 × *g* for 60 min at 37 °C. The supernatant was collected for the determination of the [^14^C]-cholesterol content using a liquid scintillation counter.

### Molecular weight (M.W.) distribution of IB2 containing peptides estimated by gel filtration

Molecular weight distribution of IB2 containing peptides estimated by gel filtration.

IB2 contained the peptides having a molecular weight < 700 was estimated by gel filtration using Superdex Peptide 10/300 GL (GE Healthcare) and the AKTA Avant 25 system (GE Healthcare). Molecular marker is IIAEKK (M.W. 700.1), IIAE (M.W. 445.3), IIA (M.W. 316.2), IA (M.W. 201), G (M.W. 75). IB2 (0.5 mg) was dissolved in 1 mL of 150 mM NaCl, 0.02 M phosphate buffer (pH 7.5) and applied to the column. The flow rate was 2.0 mL/min. Online UV absorbance was monitored at 220 nm.

### MALDI-TOF/MS analyses

The peptide in the fraction IB2 was analyzed by MALDI-TOF mass spectrometry (AXIMA Performance, SHIMADZU).

### Chemicals

FP (Phenylalanine-proline: purity (>95%)) was obtained from Bachem AG (Bubendorf, Switzerland).

### Cholesterol absorption assay for Caco-2 cells

Caco-2 cells were grown in 48-well plastic dishes containing 0.5 mL of fetal bovine serum supplemented with DMEM for 14 days post-confluence. [^14^C]-Labeled micellar cholesterol uptake in Caco-2 cells for 120 min was measured as described previously^11^. The [^14^C]-labeled micellar solutions (0.5 mL) contained casein tryptic hydrolysate (CTH), HPHU or FP, 5 g/L respectively. The [4-^14^C]-labeled micellar solution (6 mL) was prepared at the following concentrations and mixed by sonication (Taitec, VP-5): 3.7 kBq [4-^14^C]-cholesterol (2.0 Gbq/mmol, NEN), 2 µmol/L cholesterol (Sigma Aldrich), 6.6 mmol/L sodium taurocholate (Sigma Aldrich), 20 µmol/L oleic acid (Sigma Aldrich), 0.6 mmol/L phosphatidylcholine (Sigma Aldrich), 5 µmol/L monoolein (Sigma Aldrich). The cellular protein was determined using a commercially available kit (Bio-Rad Protein Assay; Bio-Rad).

### Cell culture

Caco-2 cells were acquired from the American Type Culture Collection (Manassas, USA). The cells were maintained in Dulbecco’s modified Eagle’s medium (DMEM) supplemented with 10% fetal calf serum, 4 mmol/L l-glutamine, 50,000 IU/L penicillin, and 50 mg/L streptomycin. For experiments, cells were seeded in 6-well Transwell® plates (Corning, Inc., USA) at a density of 2 × 10^5^ cells/well and grown for 14 days post-confluence. On day 14, the medium was replaced with serum-free DMEM with or without FP. All cells were incubated at 37 °C in a humidified atmosphere of 5% CO_2_ in air.

### RNA preparation from Caco-2 cells and real-time PCR

Caco-2 cells were treated with or without 1 mM FP for 24 h. After treatment, total RNA was isolated from Caco-2 cells using a NucleoSpin® RNA Kit (MACHEREY-NAGEL, Düren, Germany) and treated with DNase I using an RNase-Free DNase set (Qiagen). The total RNA was converted to cDNA using a High-Capacity cDNA Archive Kit (Applied Biosystems). Real-time PCR was performed using a StepOnePlus^TM^ Real-time PCR system (Applied Biosystems) and SYBR^®^ Premix Ex Taq (TAKARA), according to the manufacturers’ protocols. The following primers were used: ABCA1, TGCTGCATAGTCTTGGGACTC (forward) and ACCTCCTGTCGCATGTCACT (reverse); NPC1L1, AGAGGAAATGGGAGGCTTC (forward) and GGTGCGATTGATCTCGTCTT (reverse); ACAT2, CCGGAAGATGTGTCTGAGGT (forward) and CACCCACACTGGCTTGTCTA (reverse); MTP, ACCTGCAGACGTATTCATTC (forward) and CCCAGCTAGGAGTCACTGAGA (reverse); ApoAI, GAAAGCTGCGGTGCTGAC (forward) and GCCAGGTCCTTCACTCGAT (reverse); SREBP2, AGCCCTCTATTGGATGATGC (forward) and AAGAATCCGTGAGCGGTCTA (reverse); LXRα, ACTGCCCACCCAGGAAGT (forward) and CCCAGCCACAAGGACATC (reverse); LXRβ, TTCACCTACAGCAAGGACGA (forward) and GAACTCGAAGATGGGGTTGA (reverse); and for 18 S ribosomal RNA, CTCAACACGGGAAACCTCAC (forward) and CGCTCC ACCAACTAAGAACG (reverse).

### Western blot analysis of Caco-2 cells

Caco-2 cells were treated with or without 1 mM FP for 24 h. After treatment, cells were washed twice with ice-cold phosphate-buffered saline and lysed with 100 μL of NUN buffer containing 0.33 M NaCl, 1.1 M urea, 1% Nonidet P-40, 25 mM HEPES (pH 7.6), and proteinase inhibitors (Roche, Basel, Switzerland) by direct addition to the plate. Cell lysates were collected and cleared by centrifugation at 20,000 × *g* for 15 min at 4 °C. The supernatants were collected as whole-cell lysates. The cell lysates were separated by 8% SDS-PAGE and then transferred to a polyvinylidene difluoride membrane (Merck Millipore, Darmstadt, Germany). After blocking, the membranes were incubated with the following specific antibodies: anti-ABCA1 (sc-58219, Santa Cruz Biotechnology), anti-NPC1L1 (sc-166802, Santa Cruz Biotechnology), anti-LXRα (NBP1–77106, Novus Biologicals), anti-LXRβ (NBP100–74457, Novus Biologicals), and anti-β-actin (sc-47778, Santa Cruz). Immunoblot analyses were performed using an ImmunoStar^®^ LD system (Wako Pure Chemical Industries).

### Transient transfections and luciferase assay using Caco-2 cells

Caco-2 cells were transfected with 2 μg of the human pGK3-ABCA1-Luc plasmid containing the promoter region (−928 to + 101 bp) of the human ABCA1 gene and with 0.5 μg of pPGK β-galactosidase plasmid as an internal control. Transfection was allowed to proceed for 48 h in serum-free Opti-MEM I (Life Technologies, Carlsbad, USA) using Lipofectamine^®^ LTX&PLUS^TM^ (Life Technologies). After transfection, cells were incubated with 1 mM FP for 12 h. The cells were then lysed with reporter lysis buffer (Promega, Madison, USA). Luciferase activity was measured using a luciferase assay system (Promega) and a Fluoroskan Ascent FL instrument (Labsystems) according to the manufacturers’ instructions. β-Galactosidase activity was measured using a β-Galactosidase Enzyme-linked Immunosorbent Assay (ELISA) Kit (Promega) and was normalized to luciferase activity.

### Animals and diets

Male Wistar rats (Japan SLC, Hamamatsu, Japan) were used for the animal study. The rats were housed in individual cages in an environmentally controlled room maintained at 22 ± 2 °C and a 12-h cycle of light (8:00–20:00) and dark, with free access to food and water. After acclimation to a commercial nonpurified diet (MF, Oriental Yeast, Tokyo, Japan) for 3 days, 4-week-old rats weighing about 70 g were divided into 2 groups (control and FP) of 5 rats each based on body weight. Each group had free access to a high-fat high-cholesterol diet containing casein during the experimental period. The composition of high-fat high-cholesterol diets was as follows (all values are expressed as percentages) casein, 20; coconut oil, 24; corn oil, 1; cellulose, 5; AIN93 mineral mix, 3.5; AIN93 vitamin mix, 1; sucrose, 43.5; cholesterol, 1; cholic acid, 0.5; choline chloride, 0.2; dl-methionine, 0.3. The peptide FP (600 mg/kg/day) dissolved in 0.5% carboxymethyl-cellulose sodium salt (Sigma Aldrich) solution was administered orally to rats once a day at 8:00 a.m. for 14 days using a polyethylene zonde. In the experiment, body weight was measured daily and food consumption was measured every 2 days. Fecal collection (11–13 days) for the determination of fecal steroids was completed before food deprivation. At the end of the feeding period, the rats were killed under isoflurane anesthesia by drawing blood from the heart after 8 h of starvation. The total RNA for hepatic or jejunal mRNA expression analyses was isolated from the rat liver or jejunum using the RNeasy Mini Kit (Qiagen, Hilden, Germany) and treated with DNase I using an RNase-Free DNase Set (Qiagen). Livers for lipid analyses were rinsed with saline and stored at −20 °C until analysis. Serum, liver, and fecal lipids were determined as described previously^11^. The Ethics Committee on Animal Experiments at Gifu University approved all experimental protocols (permit number:15125). All experiments used in this study were performed by the experimental guidelines and regulations of Gifu University.

### RNA preparation from rat tissues and real-time PCR

The total RNAs isolated from the rat liver, jejunum, and ileum were converted to cDNA using a High-Capacity cDNA Archive Kit (Applied Biosystems, Foster City, CA, USA). Real-time PCR was performed using a StepOnePlus^TM^ Real-time PCR system (Applied Biosystems) and SYBR^®^ Premix Ex Taq (TAKARA, Shiga, Japan), according to the manufacturers’ protocols. The following primers were used: LDLR, CACTGTGGCAGTAGTGAGTG (forward) and GGCTACCGTGAATACAGGAG (reverse); HMGCR, GACCAACCTTCTACCTCGCG (forward) and ACAAACTCACCAGCCATCACAGT (reverse); CYP7A1, CTGTCATACCACAAAGTCTTATGTCA (forward) and ATGCTTCTGTGTCCAAATGCC (reverse); ApoAI, CTGGGTTCAACTGTTGGTCG (forward) and GGGCTGCATCTTCTGTTTCA (reverse); ABCA1, CAGGTATCGGGGTCCAATG (forward) and CATTATGCTGGGGACAGACT (reverse); NPC1L1, ATCTTAACTGTCGGATCCACAAAAA (forward) and AACCTGATGGCATTGTGAGACAT (reverse); and β-actin, CTCTCAGCTGTGGTGGTGAA (forward) and AGCCATGTAGCCATCC (reverse).

### Cholesterol absorption in *PepT1* knockout (KO) and wildtype (WT) mice *in vivo*

Cholesterol absorption in rats *in vivo* was measured by previously described method^11,38^. *PepT1* knockout (KO)mice and wild type (WT) mice (Trans Genic Inc., Japan) were used for the animal study. The mice were housed in individual cages as shown in rats experiments. After acclimation to a commercial nonpurified diet (MF, Oriental Yeast, Tokyo) for 3 d, 8-week-old mice weighing about 25 g were divided into 4 groups (WT control group: WTC, WT FP group: WTFP, KO control group: KOC, KO FP group: KOFP) of 8 or 9 mice each based on body weight. Each group had free access to a high-fat high-cholesterol diet containing casein during the experimental period. The composition of high-fat high-cholesterol diets was the same as rat experiment. The peptide FP (600 mg/kg/day) dissolved in 0.5% carboxymethyl-cellulose sodium salt (Sigma Aldrich) solution was administered orally to mice once a day at 8:00 a.m. for 14 days using a polyethylene zonde. In the experiment, body weight was measured daily and food consumption was measured every day. At the end of the feeding period, the mice received the test solutions by intragastric intubation with a polyethylene catheter. One hour later, the mice were killed under isoflurane anesthesia by drawing blood from the heart. Blood was collected by cardiac puncture for separation of the serum, and the liver and intestines were quickly excised. The liver was rinsed with ice-cold saline, and the luminal contents of the small intestine were flushed out with ice-cold saline. The test solutions consisted of 1 mmol/l monoolein (Sigma), 5 mmol/l taurocholic acid (Sigma), 37 kBq [1,2^−3^H]-cholesterol (1972.1 GBq/mmol, NEN) in 0.5 ml of 15 mmol/l phosphate buffer (pH 7.4). All of the solutions were emulsified by sonication (Ultrasonic Homogenizer, Model VP-5, Taitec, Tokyo). The [^3^H]-cholesterol incorporated into the serum, liver, and intestine was extracted with hexane after saponification with KOH-ethanol. Aliquots of the organic extract were used for scintillation counting. Serum and liver lipids were determined as described previously^11^. The Ethics Committee on Animal Experiments at Gifu University approved all experimental protocols (permit number:H30–192). All experiments used in this study were performed by the experimental guidelines and regulations of Gifu University.

### Statistical analyses

Values are expressed as means ± SEM. The statistical significance of differences were evaluated by Student’s *t*-tests^39^ and Tukey’s tests^40^. Differences were considered significant when P < 0.05.

## Supplementary information

Supplementary Table 1

## Data Availability

The data supporting the findings reported herein are available on request, from the corresponding author.
