# Supplementary material for: Identification of a novel cholesterol-lowering dipeptide, phenylalanine-proline (FP), and its down-regulation of intestinal ABCA1 in hypercholesterolemic rats and Caco-2 cells
Source: Sci Rep. 2019 Dec 19;9:19416. doi: 10.1038/s41598-019-56031-8 (PMC6923426; doi:10.1038/s41598-019-56031-8)
Supplement: Supplementary file 1 — Supplementary Table 1 [file 41598_2019_56031_MOESM1_ESM.pdf]

Supplementary Information

Identification of a novel cholesterol-lowering dipeptide, phenylalanine-proline (FP), and its down-regulation of intestinal ABCA1 in hypercholesterolemic rats and Caco-2 cells

Arata Banno[1+], Jilite Wang[2,+], Kenji Okada[1], Ryosuke Mori[1], Maihemuti Mijiti[1] and Satoshi Nagaoka[1,*]

[1]Department of Applied Life Science, Faculty of Applied Biological Sciences, Gifu University, 1-1 Yanagido, Gifu 501-1193, Japan, [2]Department of Agriculture, College of Hetao, Bayannur, 015000, China

[+]these authors contributed equally to this work.

*corresponding: nagaoka@gifu-u.ac.jp

Supplementary Table 1

	WT control (WTC)	WTFP	KO control (KOC)	KOFP
Body weight (g, 14d)	22.14 ± 0.62	24.34 ± 0.90 *	22.75 ± 0.41	24.39 ± 0.50 #
Food Intake (g/14d)	34.70 ± 1.64	37.34 ± 2.90	33.57 ± 1.67	39.57 ± 1.71 #
Liver weight (g/100 g B.W.)	4.69 ± 0.18	4.20 ± 0.12 *	5.43 ± 0.31	5.30 ± 0.23
Liver total lipids (μmol/g of liver)	245.4 ± 36.3	100.1 ± 14.1 **	228.1 ± 20.4	244.2 ± 28.6

Supplementary Table 1. Effects of FP on metabolic parameters in PepT1 knockout (KO) mice and wildtype (WT) mice fed a high-fat high-cholesterol diet.

Values are means ± SEM, n = 8 or 9. Statistical significance compared with the control group (WTC vs WTFP) by Student's t-test (*P<0.05, **P<0.01). Statistical significance compared with the control group (KOC vs KOFP) by Student's t-test (#P<0.05) .
